# Supplementary material for: Automated single-molecule imaging in living cells
Source: Nat Commun. 2018 Aug 3;9:3061. doi: 10.1038/s41467-018-05524-7 (PMC6076334; doi:10.1038/s41467-018-05524-7)
Supplement: Supplementary file 1 — Supplementary Information [file 41467_2018_5524_MOESM1_ESM.pdf]

Automated single-molecule imaging in living cells

Yasui et al.

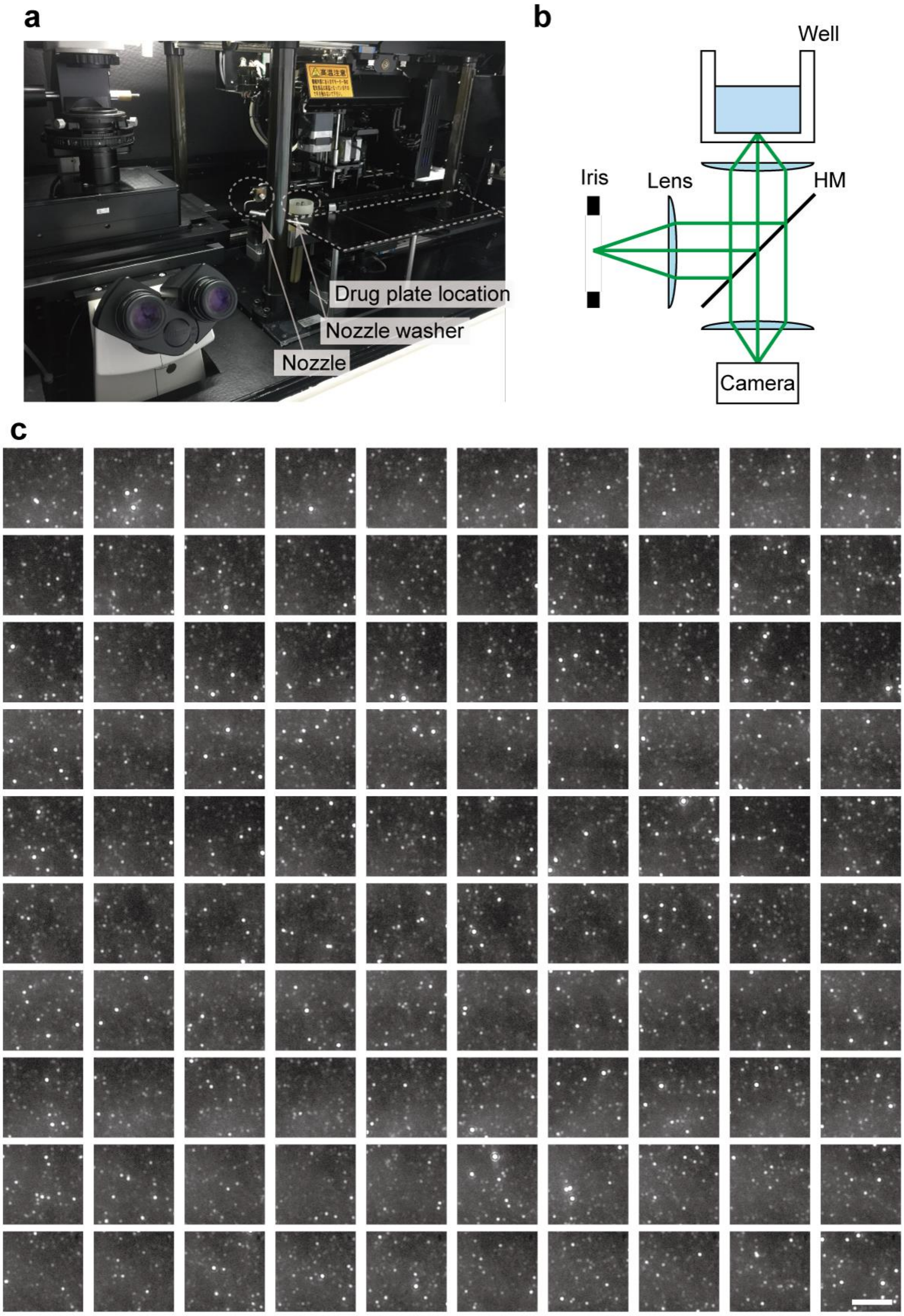

Supplementary Figure 1. AiSIS setup and autofocusing. a, Photograph of the AiSIS setup. The microscope is located on the left side. The drug plate, dispenser nozzle, and nozzle washer are

located on the right side. **b**, Optics used for automatic focusing. HM: half mirror. The positions of the iris, upper coverslip surface, and detector plane of the camera are conjugated. Thus, when the coverslip surface is shifted from the in-focus position, the image of the iris taken by the camera is blurred. **c**, Success rate of automatic focusing. Autofocusing was performed 100 times, and gold particles illuminated with a 561 nm laser were used to determine whether the focus was on the glass surface. The exposure time was 33 ms, and 100 frames were averaged. Since no bright spots were blurred in the 100 images, autofocusing was considered completely successful. Scale bar: 5 μm.

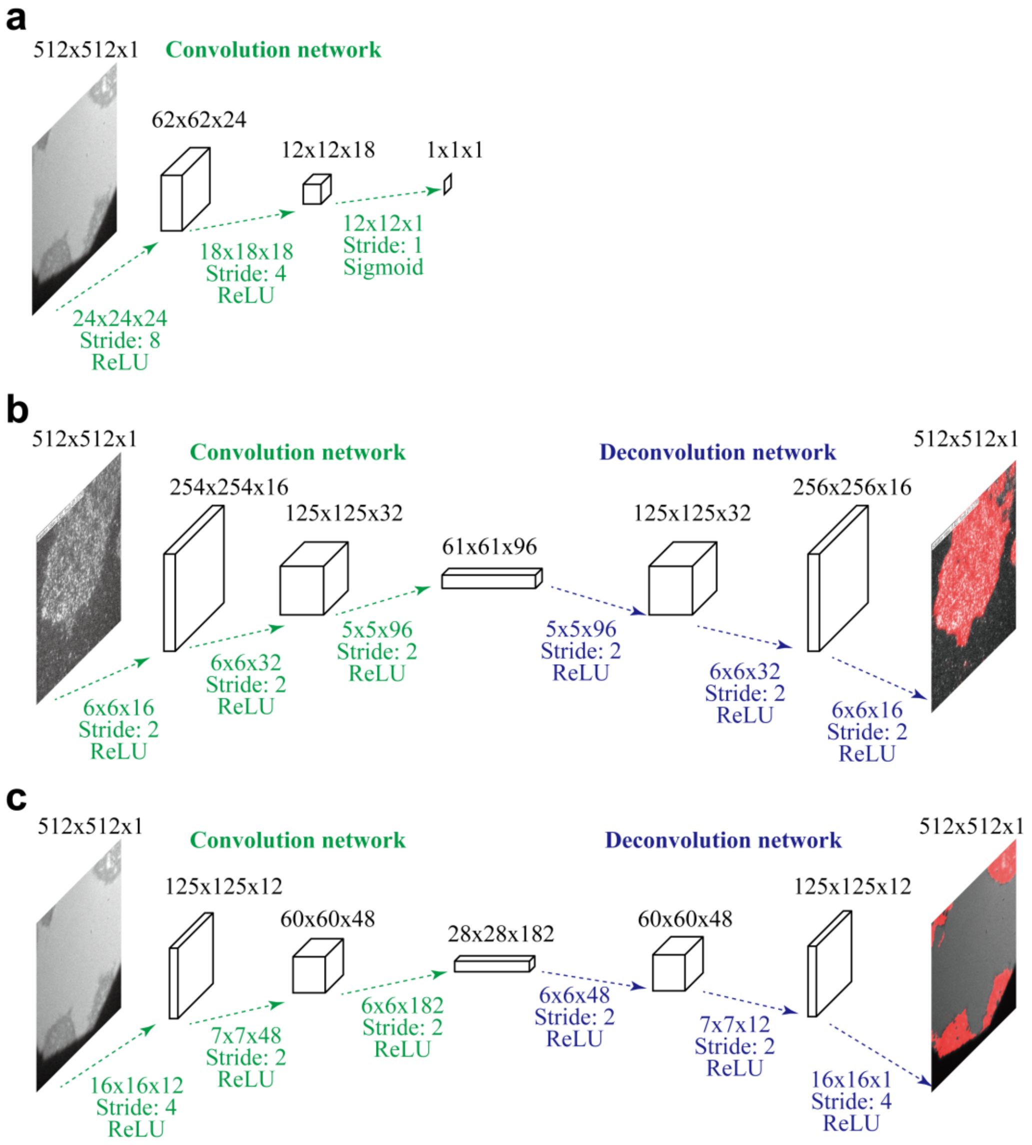

Supplementary Figure 2. Structure of the neural network. a, Coarse autofocusing. **b**, Cell searching. **c**, Cell region detection. The rectangular parallelepipeds indicate a chunk of data, and the three numbers above indicate the widths, heights and number of features quantities. Green and blue dotted arrows indicate the flow of information in convolution and deconvolution, respectively. The numbers below the arrows indicate the width x height x number of features quantities in the output. The stride and activation functions are provided below the values. The required number of training

data points was 400, 40 and 200 for the coarse autofocus, cell search, and cell region detection, respectively (See also Supplementary Figure 14).

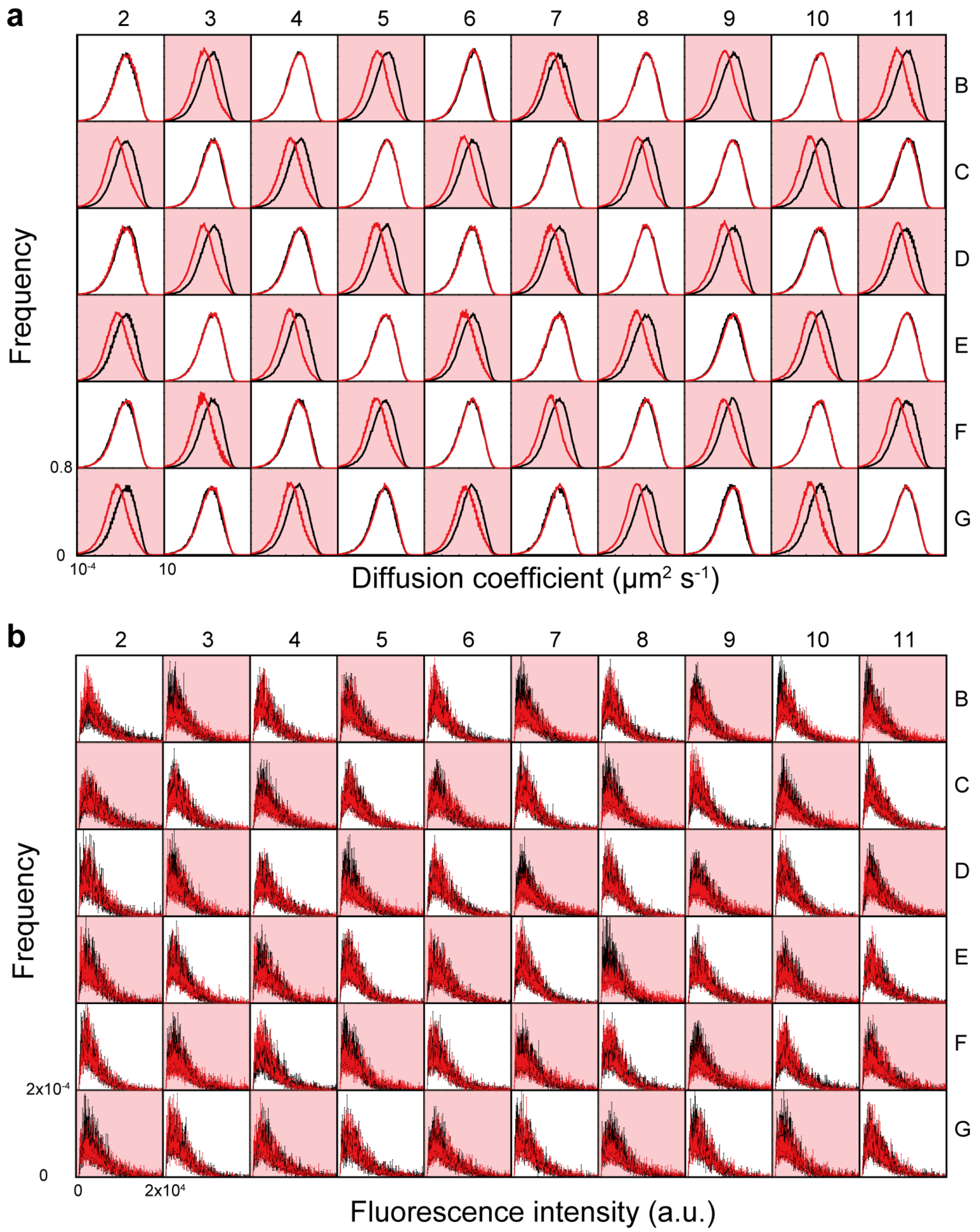

Supplementary Figure 3. Diffusion coefficients and fluorescence intensities of EGFR in each well. a, Diffusion coefficients. **b**, Fluorescence intensities. The central 60 wells of a 96-well plate were used for the single-molecule observations. Numbers (2-11) and letters (B-G) indicate the

position of each well in the plates. White and red areas indicate wells that received mock or 60 nM EGF solution, respectively. Distributions before (black) and after (red) the addition of the solutions are shown. Error bars: SE.

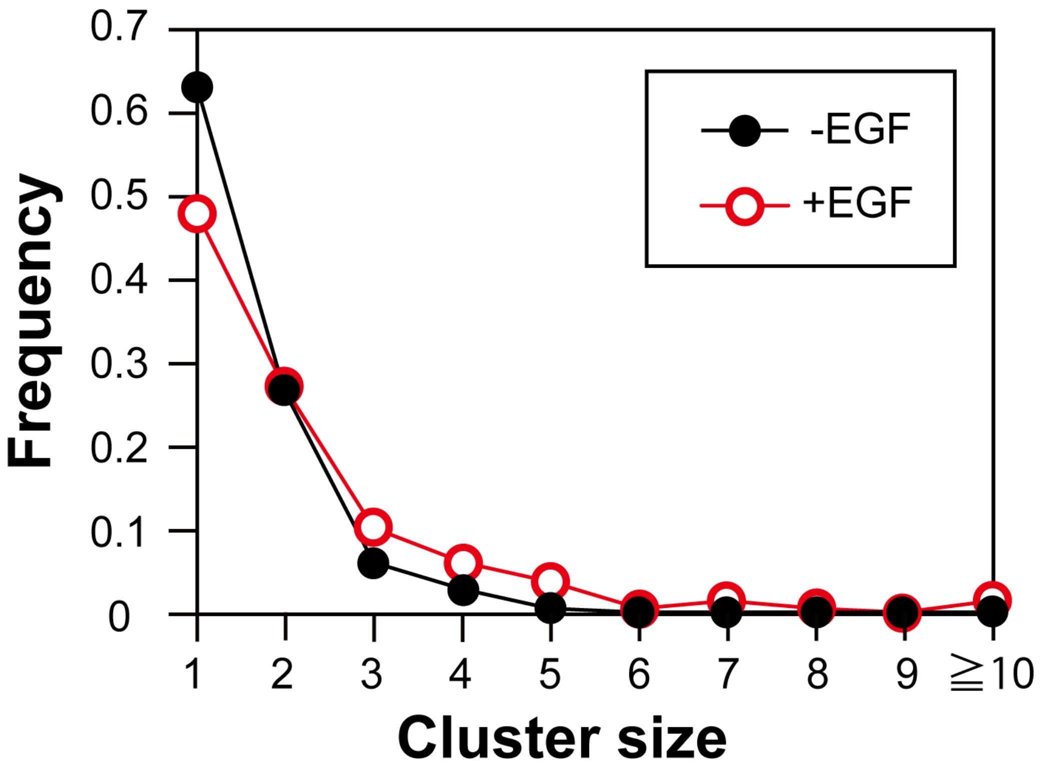

Supplementary Figure 4. Distribution of EGFR cluster size. The cluster size histogram was calculated from the brightness distribution of fluorescence spots in the first frame (Figure 2d) using equation (12). Black and red circles indicate the oligomer size distribution after the addition of 60 nM EGF and mock solutions, respectively. The total number of cells was 144 (mock solution, -EGF) and 147 (+EGF), and the total number of spots was 55,339 (mock solution) and 61,711 (+EGF). The global parameters of μ and σ were 3836 and 1395 (a.u.), respectively. The average ± SD values of the cluster sizes were 1.5 ± 0.9 and 2.1 ± 1.7 for the mock solution and 60 nM EGF, respectively.

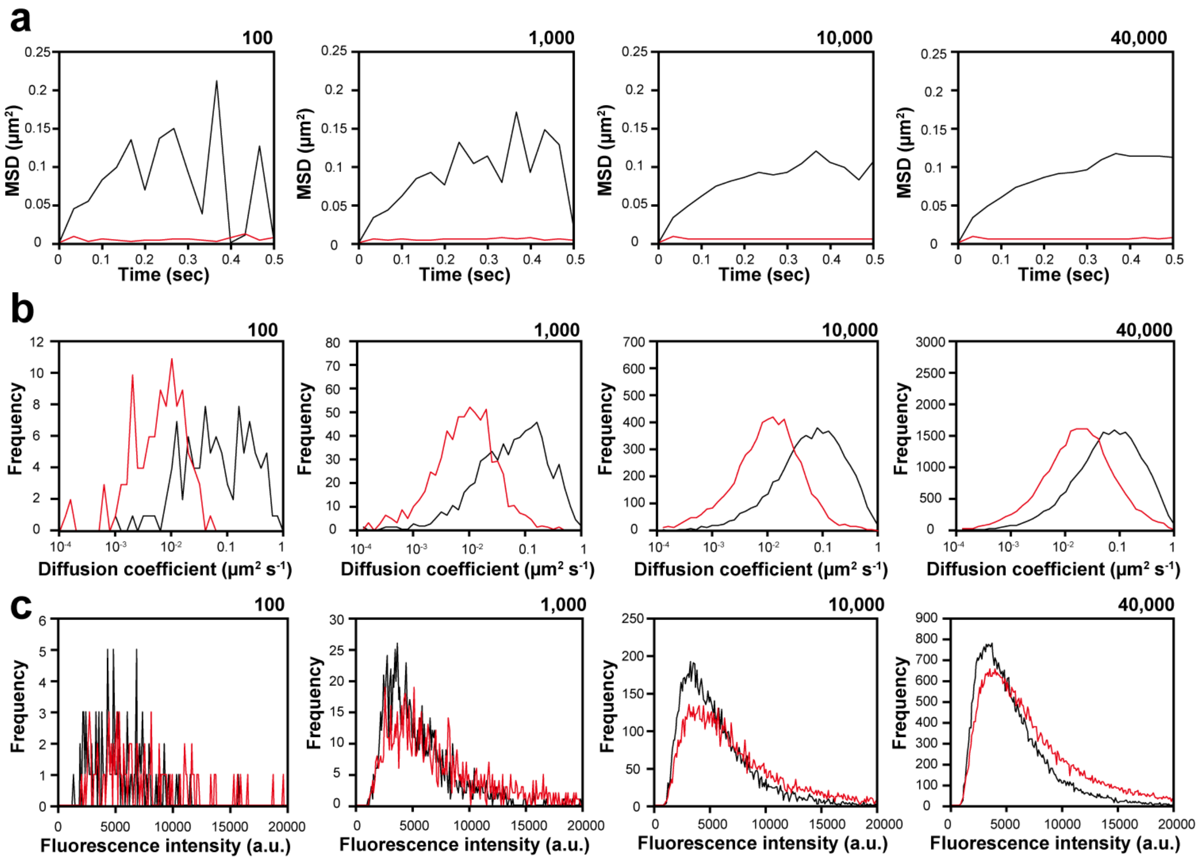

Supplementary Figure 5. Improvement of the statistical quality. Quality of the MSD (**a**), the diffusion coefficient histogram (**b**), and the intensity histogram (**c**) depends on the number of molecules. Number of fluorescent spots from left to right: 100, 1000, 10000, and 40000. Black and red lines indicate before and after the addition of 60 nM EGF, respectively.

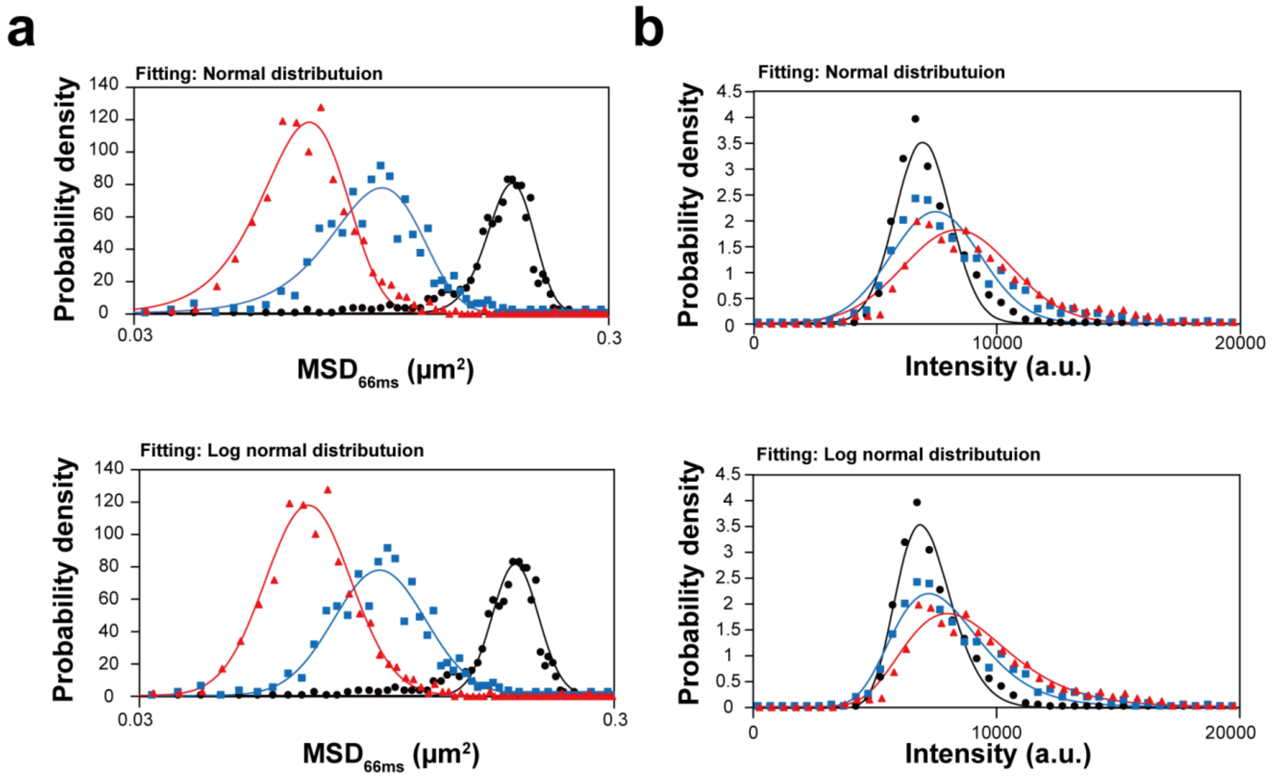

Supplementary Figure 6. Cell-to-cell variations of single-molecule parameters. MSD (**a**) and intensity (**b**) distributions under different EGF concentrations. The solid curves in the upper and lower figures are obtained by least square fitting. The deviation between data and the fit was assumed to obey normal distribution or log-normal distribution as indicated in the graph. The parameters obtained with the log-normal distribution showed a slightly higher log-likelihood than that obtained with the normal distribution. 7863 / 8088 (MSD) and -19592 / -19267 (intensity) for normal / log-normal distribution. The colors black, blue, and red indicate EGF concentrations of 0, 6, and 60 nM, respectively. The source of the data is the same for Figure 2e.

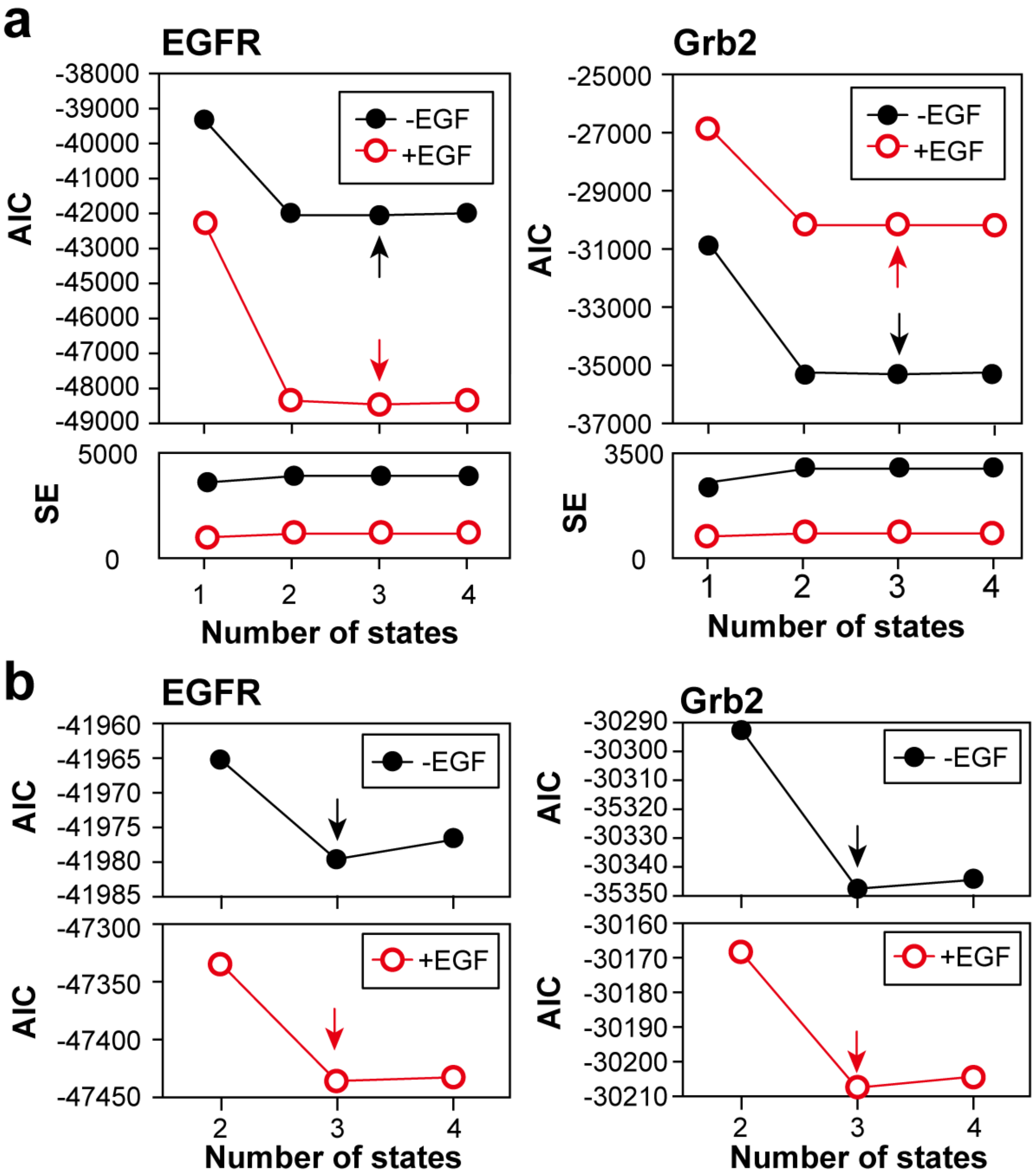

Supplementary Figure 7. AIC values obtained with different numbers of motional states. a, Minimum values were typically shown for 3 states (arrows). Left and right panels represent EGFR and Grb2, respectively. AIC values before (black) and after (red) the addition of 60 nM EGF are indicated. The upper and lower panels show the mean and SE, respectively. **b,** Sub-regions (**a**)

including AIC values for 2 to 4 states are magnified to discriminate the state with the minimum value.

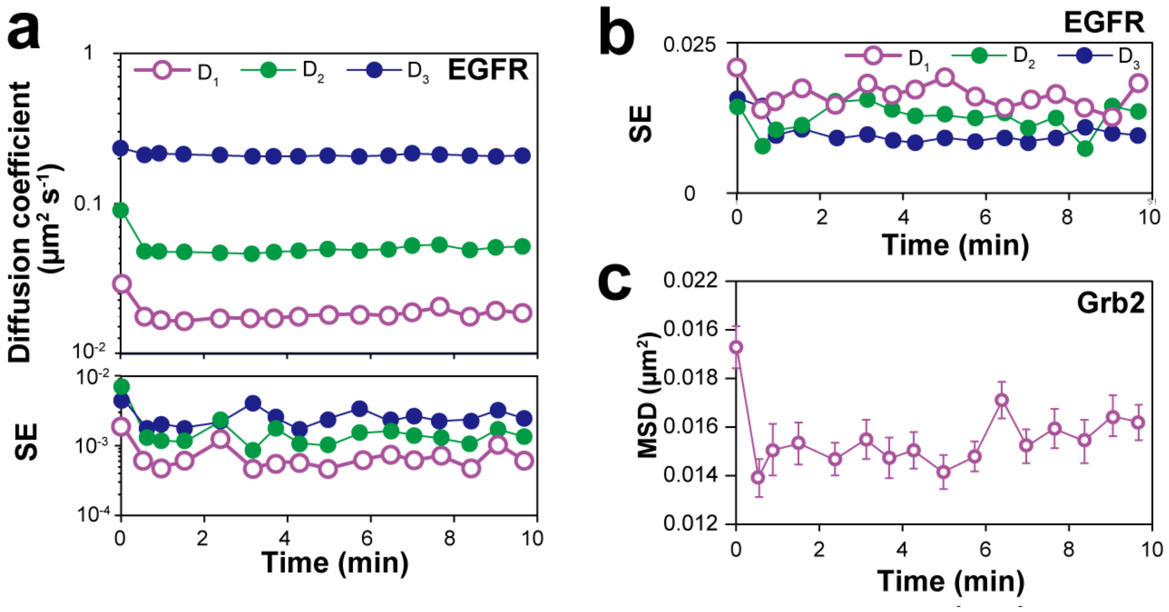

Supplementary Figure 8. Time course of the molecular behaviors of EGFR and Grb2. a, Diffusion coefficients of EGFR. **b**, SE of the EGFR fractions shown in Figure 5a. **c**, MSD at Δt = 66 ms of Grb2. Magenta, green and blue circles (**a**, **b**) indicate the immobile, slow-mobile, and fast-mobile states, respectively. Error bar: SE.

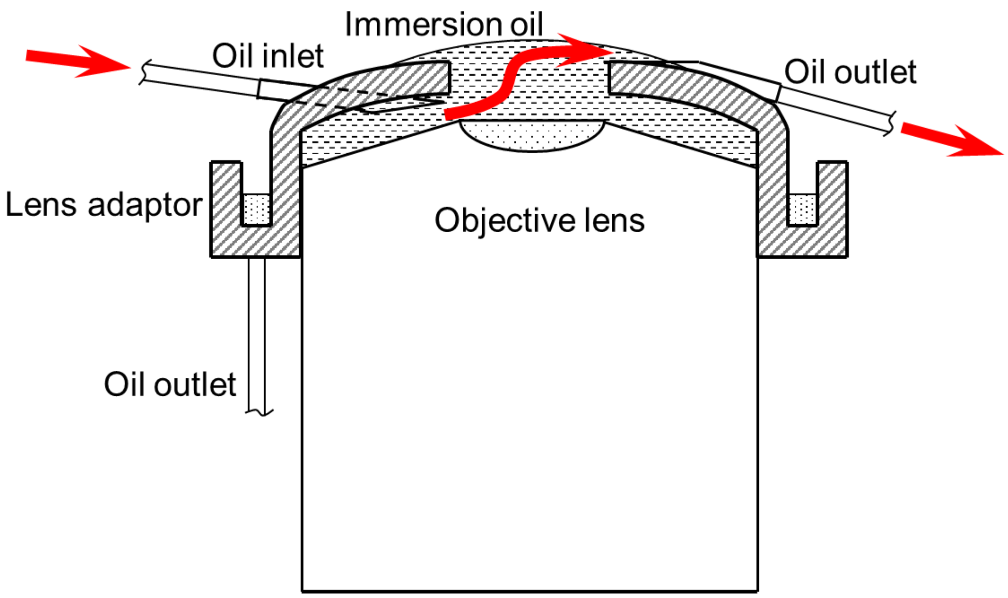

Supplementary Figure 9. Cross section of the objective lens adaptor of the immersion-oil feeding system. The oil flow is introduced from the inlet to the outlet along the path of the red arrows. An appropriate amount of oil is always pooled in the space between the lens and coverslip.

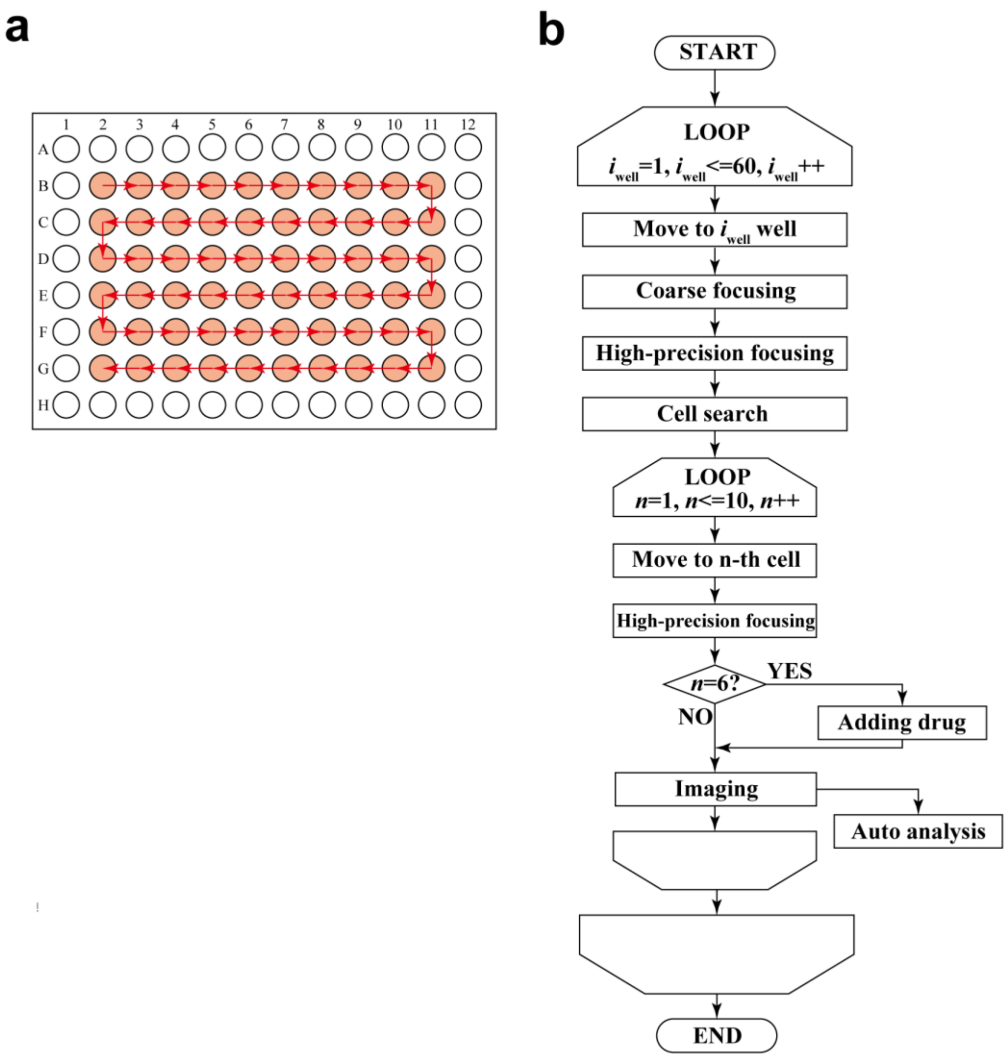

Supplementary Figure 10. AiSIS experimental procedure. a, Scanning direction in a 96-well plate. During the experiment, the microscope stage scanned 60 wells of a 96-well plate (without the 36 peripheral wells). As shown in Figure 1b, up to 10 cells suitable for single-molecule imaging were automatically searched in each well. **b**, Flowchart of the automated single-molecule imaging. After moving to a well (i_{well}), the surface of the bottom glass is focused by the following two-step procedure: coarse focusing and high-precision focusing. Then, cells with an appropriate fluorescent spot density are searched up to a preset number (10 cells in the case of Figure 1b) using the learned

neural network. Single-molecule imaging is sequentially performed for half of the selected cells before and after the ligand/mock solution is added (5 cells under each condition). Using the acquired images, single-molecule tracking and statistical analyses are performed.

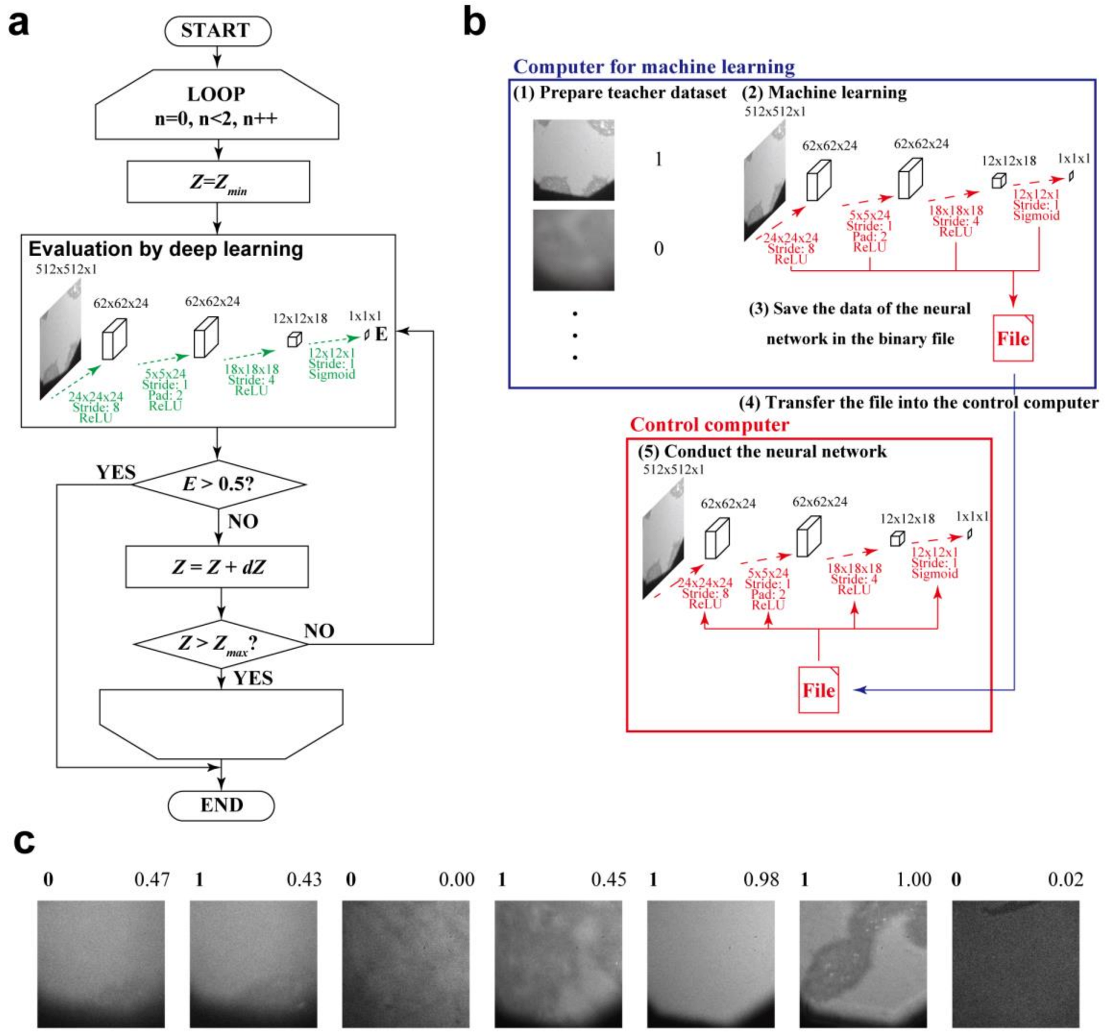

Supplementary Figure 11. Coarse autofocusing. a, Flowchart of the procedure. In advance, the neural network learned both in- and out-of-focus images of the iris. After the microscope stage is set at the target well, the objective lens scans a predefined Z range of 750 µm and evaluates the iris image using deep learning. The evaluation value is between 1 and 0, depending on whether the obtained images are judged as in-focus or out-of-focus. The procedure continues until the evaluation value is greater than 0.5 and stops if it fails twice. Z_{min} and Z_{max} represent the minimum and maximum objective positions, respectively, and dZ represents a stepping height of 2.5 µm. **b**,

Machine learning and subsequent autofocusing. Training data for in- and out-of-focus images are prepared and used for machine learning. Adaptive moment estimation (Adam) is applied to the process using Python's Chainer library. The neural network optimizes the parameters, such as the number and type of layers, activation functions, the convolution/deconvolution weight and bias values, of each layer and saves them in a binary file. During the experiment, the file is loaded by control software programmed using C ++ and CUDA on a PC equipped with GPU (NVIDIA Quadro 4000) for AiSIS control. **c**, In- and out-of-focus images of the iris and cells used as training data are shown. Numbers in bold at the upper left are the assigned values for training, with 1 or 0 assigned to images obtained less or more than 4 µm from the in-focus position, respectively. Numbers in the narrow region in the upper right of the images denote evaluation values between 0 (not focused) and 1 (focused).

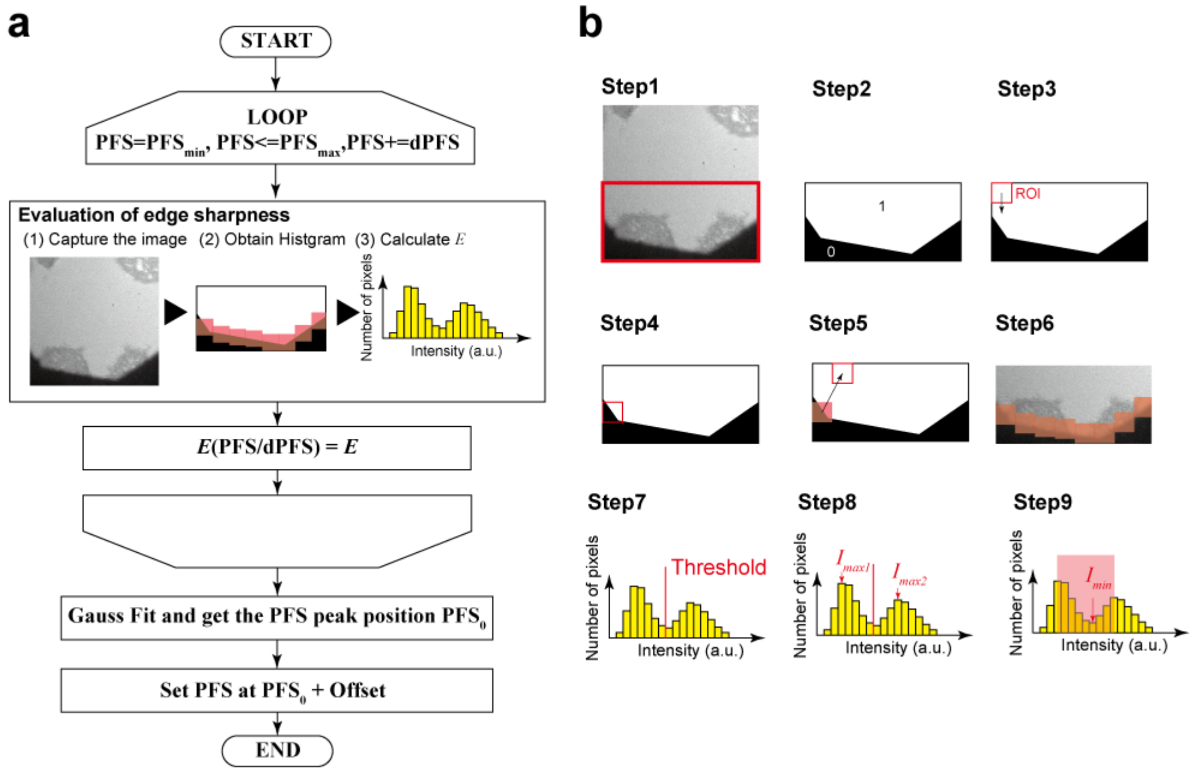

Supplementary Figure 12. High-precision autofocusing. a, Flowchart of the procedure. The objective position is first set to the initial value (PFS_{min}) and gradually moved upward discretely (step size: dPFS). Evaluation of the objective position on the acquired image is performed at each step by referencing the sharpness of the iris edge. Gaussian fitting of the brightness histogram is used to calculate the evaluation value E as described in (**b**). Finally, the objective position is moved to the position with the highest E and then fine adjusted with an offset that compensates for the position difference from the visually determined in-focus position. **b**, Algorithm used to evaluate the histogram of the pixel intensity around the iris edge. Step 1: The lower half of the iris aperture image is spliced (red boxed region). Step 2: The cropped image is binarized using the Otsu method. Step 3: The ROI (21 x 21 pixels) is moved downward. Step 4: When the black region overlaps the

ROI by more than 50%, the pixel intensities in the ROI are added to the histogram. Step 5: The ROI returns to the top of the region and shifts to the adjacent row. Step 6: Steps 3-5 are repeated until the ROI reaches the right end. Step 7: The valley between the two peaks in the brightness histogram of the ROIs in the original image (Step 6) is determined using the Otsu method. The histogram is smoothed over 20 bins. Step 8: The positions of both peaks are calculated, and the intensities are designated I_{max1} and I_{max2}. Step 9: The intensity at the bottom of the valley is calculated, and the intensity is designated I_{min}. Then, the evaluation value is calculated as $E = N(I_{max1}) \cdot N(I_{max2}) / N(I_{min}^2)$, where $N(I)$ is the value of the histogram at intensity I.

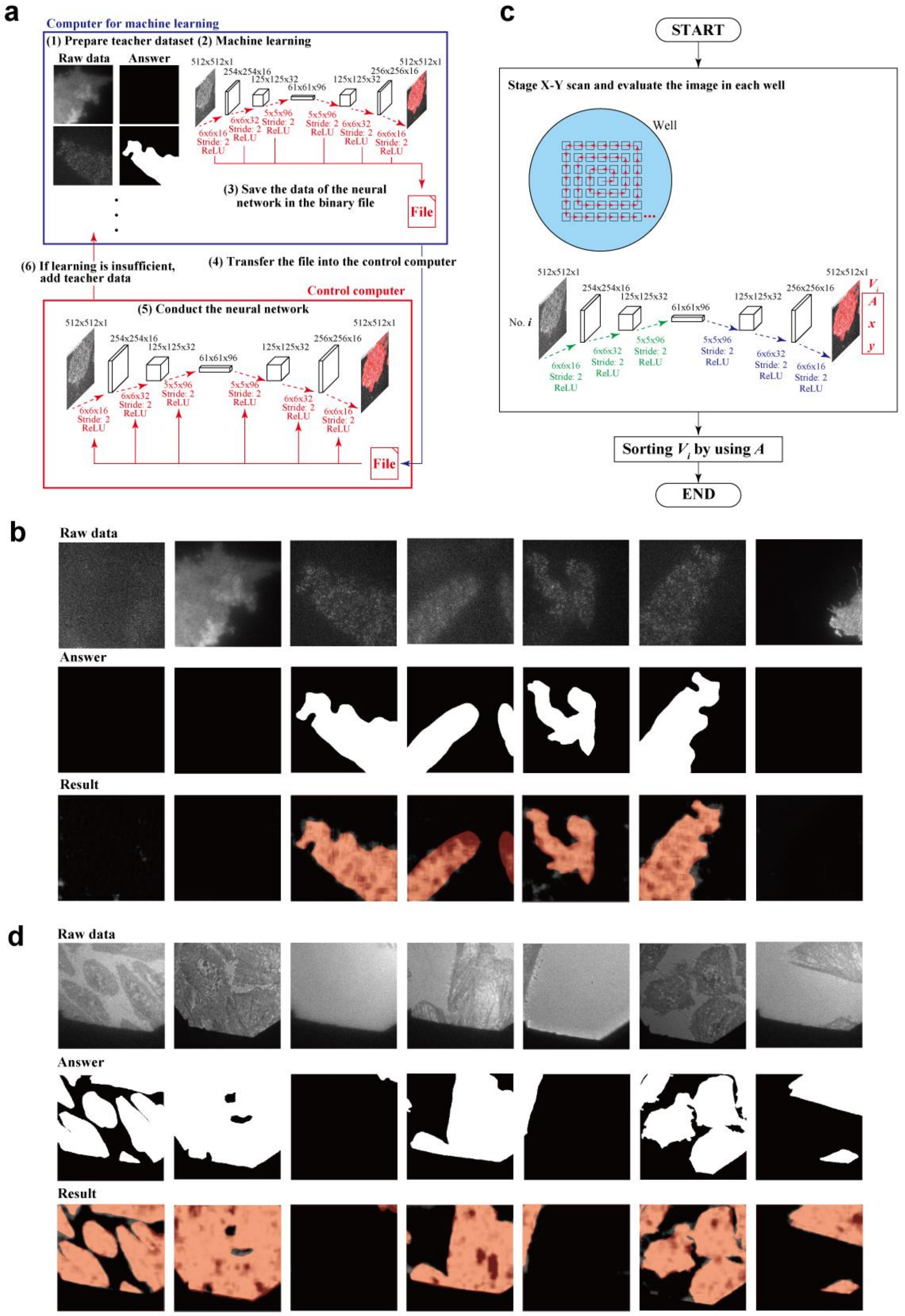

Supplementary Figure 13. Cell searching algorithm. a, Machine learning and subsequent cell

searching. Cells with various expression levels of EGFR-GFP are captured by single-molecule imaging in advance. Cell regions with suitable fluorescent spot densities are painted in white, and the processed images (middle row in (**b**)) are used as training data. The neural network consists of three convolution and three deconvolution layers. The layer parameters optimized by learning are saved in a binary file. **c**, Flowchart of the cell searching procedure in each well. Cells are searched by scanning the microscope stage along the direction indicated by the red arrows in the blue circle, and 225 (= 15 x 15) snapshots of the fluorescence images are acquired. Among the acquired images, regions with suitable EGFR-GFP expression are determined by the learned neural network. A dataset of (A_i, x_i, y_i) is assigned to the i-th largest region with area A and centroid (x_i, y_i). Then, image acquisition is initiated according to the order of Vi. **b** and **d**, Raw data (top row), training data (middle row), and output results from the learned neural network (bottom row) are shown for a single molecule (**b**) and SRIC images (**d**). The output result is overlaid on the training data in red for comparison of these images.

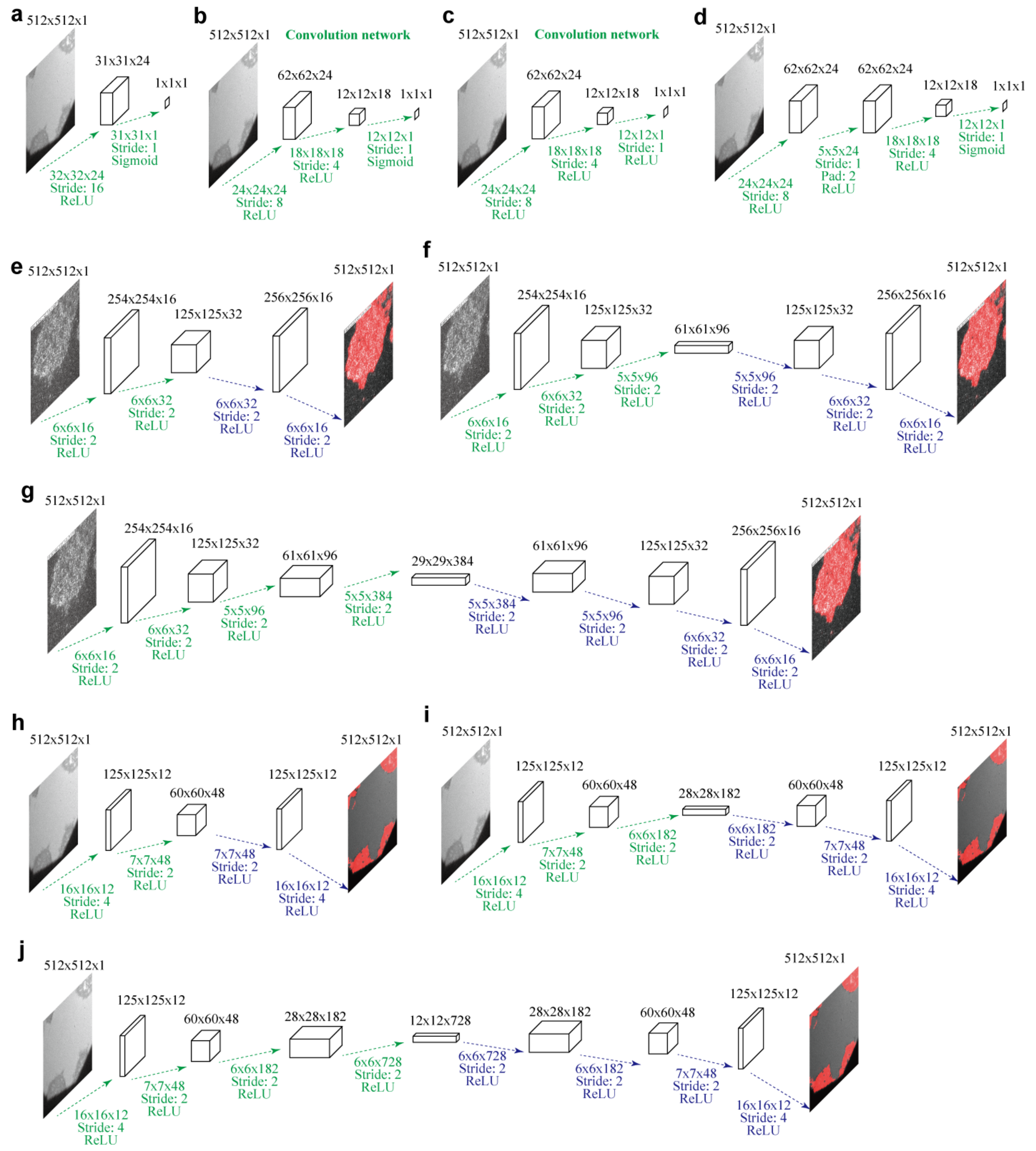

Supplementary Figure 14. Neural networks prepared to verify the optimum number of layers. **a**-**d**, Coarse autofocusing. **e**-**g**, Cell searching. **h**-**j**, Cell region detection. The number of layers for convolution/deconvolution is two (**a**, **e** and **h**), three (**b**, **f**, and **i**), and four (**c**, **g** and **j**). The rectangular parallelepipeds indicate chunks of data. The three numbers above the chunks indicate

the widths, heights and number of features quantities. The numbers below the arrows indicate the width x height x number of features quantities in the output. The stride and activation functions are provided below the values. The green and blue dotted arrows indicate the flow of information in convolution and deconvolution, respectively.

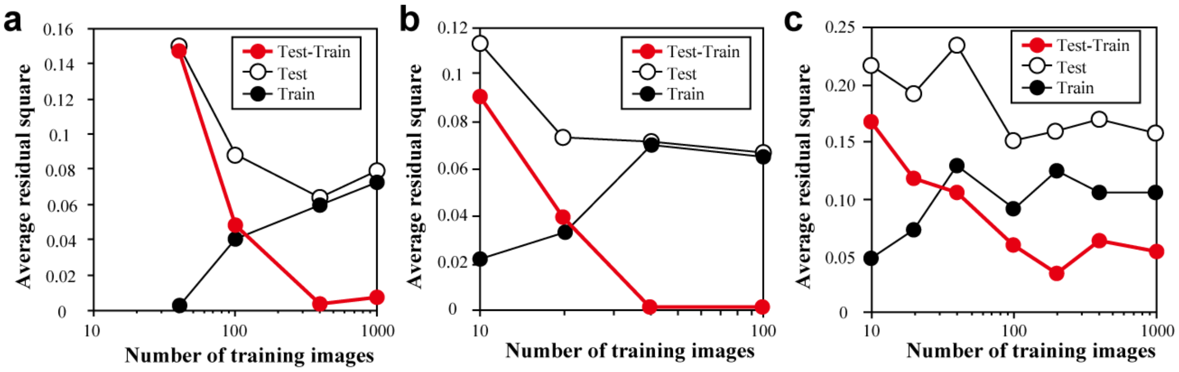

Supplementary Figure 15. Relationship between the number of training images and overlearning. a, Coarse autofocusing. **b**, Cell searching. **c**, Cell region detection. The learning was performed 10,000 times using different numbers of training data images via adaptive moment estimation (Adam)[28]. The ARS value, which is the root mean of the squared difference between the values of corresponding pixels in the original images and the predicted results from the neural network, is equivalent to the error in the prediction. Black, open, and red circles indicate the errors obtained in prediction of the training data used for learning (Train), new test data (Test), and the difference between the two (Test-Train), respectively. Based on these results, the required number of training images was 400, 40, and 200 for coarse autofocusing, cell searching, and cell region detection, respectively.

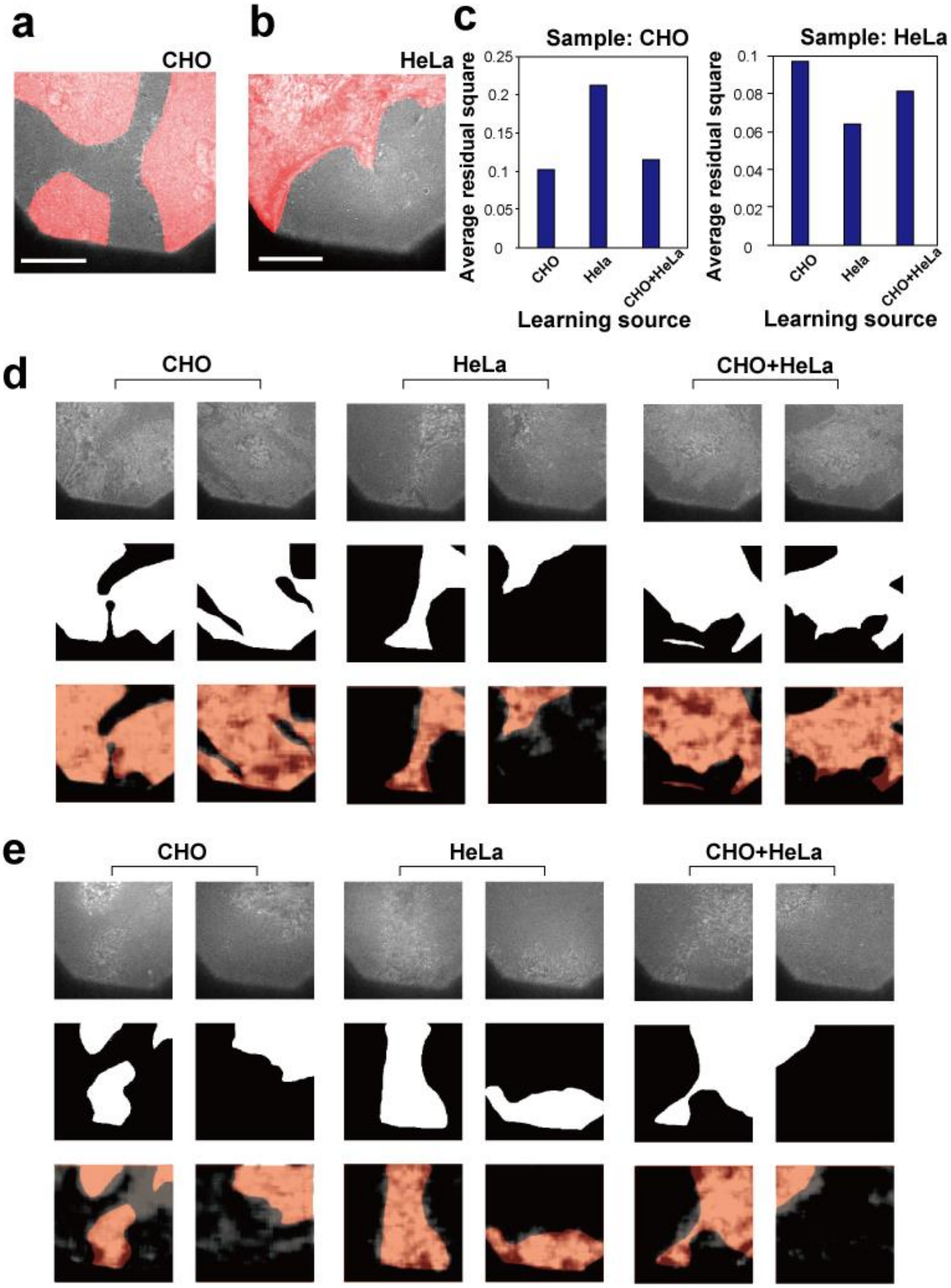

Supplementary Figure 16. Cell type-dependent learning for automatic cell region detection. a,

SRIC images of a CHO cell and **b**, HeLa cell. Cell regions are indicated in red. Scale bars, 10 µm. **c**, Comparison of ARS between the cell types (see Cell type-dependent learning in the Methods section for detail). ARS has a value > 0.5 when the network has not learned. ARS approaches 0 during learning depending on the difficulty of the tasks. Lower ARS values indicate better learning for cell region detection. ARS was observed when the algorithm was tested using images of CHO (left) and HeLa (right) cells. CHO or HeLa learning was performed using 100 images of CHO or HeLa cells. CHO + HeLa learning was performed using 50 images of CHO cells and 50 images of HeLa cells. In total, 50 images of CHO and HeLa cells were used for learning. **d** and **e**, Raw data (top), answer provided by the researcher (middle), and output results from the learned neural networks (bottom) for CHO (**d**) and HeLa (**e**) cell images. Indications denoting the type of training data (CHO, HeLa, and CHO + HeLa) have the same meaning as those in (**c**).

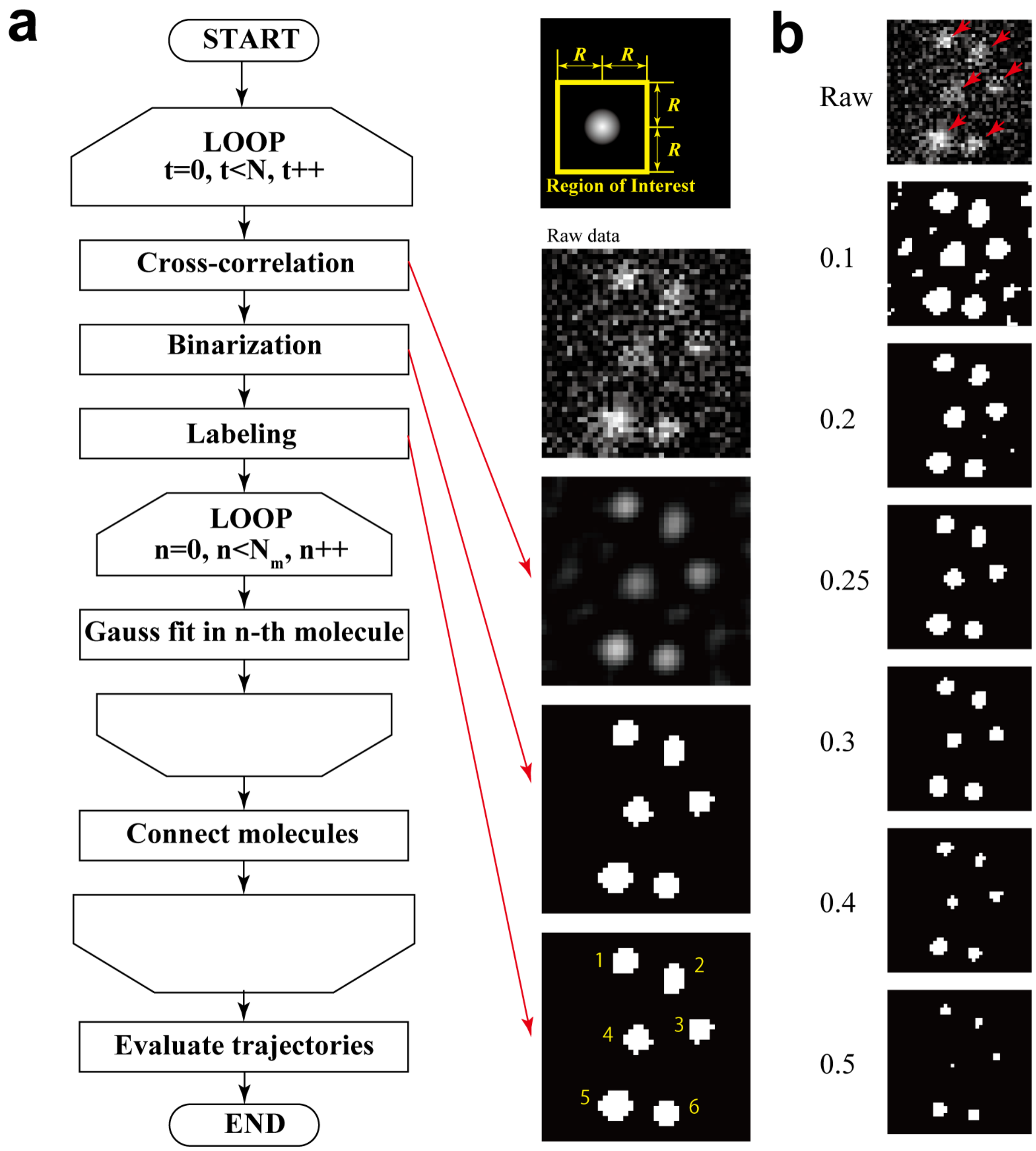

Supplementary Figure 17. Single-molecule/particle tracking. a, Flowchart of the procedure. See the Single-molecule tracking algorithm section of the Methods for details; N_m indicates the number of labeled particles. **b**, Binarized images using the indicated thresholds (values shown on the left). The red arrows indicate single molecules visually detected. The threshold of 0.25 could remove the noise (two small white spots), as shown in the binarized image using a threshold of 0.2.

Supplementary Table 1. Number of cells shown in Figure 5 at each time point.

Time (min)	Number of cells (EGFR)	Time (min)	Number of cells (Grb2)
0	50	0	45
0.56	30	0.52	21
0.90	20	0.85	24
1.49	36	1.49	32
2.35	46	2.36	43
3.11	33	3.09	28
3.68	29	3.64	30
4.25	35	4.26	32
4.96	40	4.96	36
5.71	41	5.69	37
6.41	33	6.38	32
6.99	33	6.97	34
7.64	38	7.63	33
8.37	37	8.38	31
9.02	31	9.02	31
9.64	40	9.65	36

Supplementary Table 2. Relationship among the number of layers, execution time, and precision. Errors (ARS. See Optimization of number of layers section in the Methods section) in the predictions of 100 test images were calculated based on learning 100 training data points. Letters in the column 'Neural net type' correspond to the neural networks indicated by the same letters in Supplementary Figure 14. Bold-face values indicate the network type with the lowest product of execution time and error.

	Neural net type	Number of layers	Execution time (ms)	ARS	Execution time x ARS
**Coarse Autofocusing**	a	2	1.4	0.118	**0.165**
	b	3	2	0.086	0.172
	c	3	2	0.52	1.04
	d	4	2.7	0.085	0.233
**Cell Searching**	e	2	187	0.067	12.5
	f	3	258	0.039	**10.1**
	g	4	340	0.034	11.6
**Cell Region Detection**	h	2	144	0.099	14.2
	i	3	200	0.069	**13.9**
	j	4	314	0.055	17.4

Supplementary Table 3. Fitted parameters for the calculation of confined length.

The MSD was measured for 15 time points. The value of $\log L_{log}$ was larger than the value of $\log L_{norm}$; therefore, the parameters were calculated by maximizing $\log L_{log}$ in equation (9)

Parameter	EGF-	EGF+
$\log L_{norm}$	7560	6507
$\log L_{log}$	7798	6513
L (nm)	263	626
D (μm^2/s)	0.08	0.12
σ_1	0.15	0.11
σ_2	0.18	0.11
σ_3	0.23	0.11
σ_4	0.26	0.12
σ_5	0.29	0.13
σ_6	0.31	0.14
σ_7	0.32	0.15
σ_8	0.34	0.16
σ_9	0.35	0.17
σ_{10}	0.36	0.18
σ_{11}	0.37	0.19
σ_{12}	0.38	0.2
σ_{13}	0.39	0.22
σ_{14}	0.4	0.23
σ_{15}	0.4	0.24

Supplementary Table 4. Fitted parameters for the calculation of the EC_{50} in EGF stimulation.

MSD at Δt = 66 ms was measured for ten EGF concentrations. The value of $\log L_{log}$ was larger than the value of $\log L_{norm}$; therefore, the parameters were calculated by maximizing $\log L_{log}$ in equation (15).

**Parameter**	**Value**
$\log L_{norm}$	727
$\log L_{log}$	733
h	1.0
MSD_{min} (μm^2)	0.0153
MSD_{max} (μm^2)	0.0333
EC_{50} (nM)	6.0
σ (600 nM)	0.17
σ (300 nM)	0.15
σ (60 nM)	0.19
σ (30 nM)	0.16
σ (6 nM)	0.18
σ (3 nM)	0.15
σ (0.6 nM)	0.17
σ (0.3 nM)	0.12
σ (0.06 nM)	0.24
σ (0.03 nM)	0.28

Supplementary Table 5. Fitted parameters for the calculation of EC_{50} and IC_{50}. The MSDs at Δt = 66 ms were measured for all combinations between six concentrations of both EGF and AG1478. The MSD_{min}, MSD_{max}, EC_{50} and IC_{50} were 0.0114 (μm^2), 0.0303 (μm^2), 4.65 (nM), and 2.35 (μM), respectively. The logL_{log} value was larger than the logL_{norm} value, with values of 522 and 519, respectively. Therefore, the parameters were calculated by maximizing logL_{log} in equation (17).

		**EGF concentration (nM)**					
		0.3	0.6	3	6	30	60
**Inhibitor concentration (nM)**	10000	0.03	0.13	0.09	0.18	0.19	0.16
	1000	0.16	0.03	0.06	0.1	0.08	0.05
	100	0.17	0.08	0.17	0.19	0.06	0.18
	10	0.04	0.13	0.04	0.2	0.09	0.03
	1	0.2	0.11	0.14	0.06	0.1	0.05
	0.1	0.02	0.05	0.12	0.19	0.17	0.16
