# Supplementary material for: Automated single-molecule imaging in living cells
Source: Nat Commun. 2018 Aug 3;9:3061. doi: 10.1038/s41467-018-05524-7 (PMC6076334; doi:10.1038/s41467-018-05524-7)
Supplement: Supplementary file 3 — Description of Additional Supplementary [file 41467_2018_5524_MOESM3_ESM.pdf]

Description of Additional Supplementary Files

File Name: Supplementary Movie 1
Description: **Automated single-molecule imaging system.** The automatic procedure of the single-molecule analysis is as follows: i) a multi-well plate is placed on the microscope stage, and the target well is placed above the objective lens; ii) coarse and high-precision autofocusing processes are executed on the well by moving the objective to the stage; iii) cells suitable for singlemolecule imaging are identified, and the positions of the cells are memorized up to the preset number; iv) high-precision autofocusing is conducted again, and single-molecule images are recorded at each memorized position; and v) single-molecule tracking and statistical analysis are performed. If required, a drug can be added in step iv to observe the drug's effects on the cells. A series of these operations is repeated for all wells in the plate. In this movie, the plate transport and drug addition are shown at a speed 2-times faster than real time. Autofocusing and cell searching are shown at 8- and 2-times faster speeds, respectively.

File Name: Supplementary Movie 2
Description: **Behavioral changes in EGFR molecules after EGF or mock solution addition.** During the first 2 s, all 60 wells are displayed. Then, two wells treated with mock (left) or EGF (right) solution are shown and compared for 2 s. Finally, automatic single-molecule tracking in the movies obtained from these two wells and SRIC images are shown. Scale bar: 10 µm.

File Name: Supplementary Movie 3
Description: **Autofocusing process.** The autofocusing process includes two steps. During coarse focusing, the Z stage is gradually moved 2.5 µm up from the initial position and stops where the extent of blurring converges within a criterion determined by deep learning that consists of three hidden convolution layers. The number of layers and parameters shown in Supplementary Table 2 are set to achieve both a higher precision and a shorter calculation time. After the PFS is turned on, the high-precision focusing algorithm starts to scan a limited distance along the Z axis and evaluates the sharpness of the image of the iris edge. The sharpness is calculated based on the profile of the fluorescence intensity histogram. Details are provided in the Methods section. Finally, we set the Z position with the maximum sharpness, which allowed it to shift to a previously determined offset distance to fill the gap in the in-focus position between manual and autofocusing.

File Name: Supplementary Movie 4
Description: **Automatic cell searching process.** First, the fluorescent spot images are acquired using serial X-Y scanning by snapshot (33 ms) observations via TIRFM. Then, regions similar to the prior learned images with suitable spot densities (1-3 molecules/µm 2) are selected by deep learning. The inclusion of a blurred image in the training data allows for the recognition of suitable regions, even under out-of-focus conditions. We speculate that this blur occurs due to misrecognition of the offset position by the PFS derived from the difference in the refractive index between the cell and water.

File Name: Supplementary Movie 5
Description: **Dual-color imaging of EGFR-GFP (Green) and Grb2-TMR (Magenta).** Scale bar: 5 µm.
